# A pretest‐posttest design to assess the effectiveness of an intervention to reduce HIV‐related stigma and discrimination in healthcare settings in Vietnam

**DOI:** 10.1002/jia2.25932

**Published:** 2022-07-12

**Authors:** Todd M. Pollack, Hao Thi Duong, Dang Thi Nhat Vinh, Do Thi Phuong, Do Huu Thuy, Vo Thi Tuyet Nhung, Nguyen Kieu Uyen, Vuong The Linh, Nguyen Van Truong, Kim Anh Le Ai, Nguyen Thi Ninh, Asia Nguyen, Hoang Dinh Canh, Lisa A. Cosimi

**Affiliations:** ^1^ Partnership for Health Advancement in Vietnam Hanoi Vietnam; ^2^ Department of Medicine Beth Israel Deaconess Medical Center Boston Massachusetts USA; ^3^ Baylor College of Medicine Houston Texas USA; ^4^ Vietnam Authority of HIV/AIDS Control, Ministry of Health Hanoi Vietnam; ^5^ Binh Duong Center for Disease Control Binh Duong Vietnam; ^6^ Thai Nguyen Center for Disease Control Thai Nguyen Vietnam; ^7^ Hanoi Center for Disease Control Hanoi Vietnam; ^8^ Division of Global HIV and TB Center for Global Health United States Centers for Disease Control and Prevention Vietnam Hanoi Vietnam; ^9^ Department of Medicine Brigham and Women's Hospital Boston Massachusetts USA

**Keywords:** community engagement, HIV/AIDS, key populations, people living with HIV, stigma and discrimination, Vietnam

## Abstract

**Introduction:**

Stigma and discrimination are important barriers to HIV epidemic control. We implemented a multi‐pronged facility‐level intervention to reduce stigma and discrimination at health facilities across three high‐burden provinces. Key components of the intervention included measurement of stigma, data review and use, participatory training of healthcare workers (HCWs), and engagement of people living with HIV and key populations in all stigma reduction activities.

**Methods:**

From July 2018 to July 2019, we assessed HIV‐related stigma and discrimination among patients and HCWs at 10 facilities at baseline and 9 months following an intervention. A repeated measures design was used to assess the change in stigma and discrimination among HCWs and a repeated cross‐sectional design assessed the change in stigma and discrimination experienced by PLHIV. HCWs at target facilities were invited at random and PLHIV were recruited when presenting for care during the two assessment periods. McNemar's test was used to compare paired proportions among HCWs, and chi‐square test was used to compare proportions among PLHIV. Mixed models were used to compare outcomes before and after the intervention.

**Results:**

Semi‐structured interviews were conducted with 649 and 652 PLHIV prior to and following the intervention, respectively. At baseline, over the previous 12 months, 21% reported experiencing discrimination, 16% reported self‐stigma, 14% reported HIV disclosure without consent and 7% had received discriminatory reproductive health advice. Nine months after the intervention, there was a decrease in reported stigma and discrimination across all domains to 15%, 11%, 7% and 3.5%, respectively (all *p*‐values <0.05). Among HCWs, 672 completed the pre‐ and post‐intervention assessment. At baseline, 81% reported fear of HIV infection, 69% reported using unnecessary precautions when caring for PLHIV, 44% reported having observed other staff discriminate against PLHIV, 54% reported negative attitudes towards PLHIV and 41% felt uncomfortable working with colleagues living with HIV. The proportions decreased after the intervention to 52%, 34%, 32%, 35% and 24%, respectively (all *p*‐values <0.05).

**Conclusions:**

A multi‐pronged facility‐level intervention was successful at reducing healthcare‐associated HIV‐related stigma in Vietnam. The findings support the scale‐up of this intervention in Vietnam and highlight key components potentially applicable in other settings.

## 1 INTRODUCTION

Globally, stigma and discrimination are recognized as important barriers to ending AIDS by 2030 [1]. People living with HIV face stigma and discrimination in all aspects of their lives; within their families, communities, workplace and when seeking healthcare [2]. Stigma is often intersectional; in addition to HIV‐related stigma, key populations (KPs) experience stigma related to substance use, sex work, gender identity and sexual orientation [3, 4, 5, 6]. In the healthcare setting, HIV‐related stigma affects access to and utilization of health services as well as the quality of care provided to people living with HIV [7]. Numerous studies have demonstrated the negative effects of stigma and discrimination on HIV testing, disclosure, linkage to care and adherence to antiretroviral therapy (ART) [8, 9]. The manifestations of HIV‐ and KP‐related stigma in health facilities are well documented and include a refusal to provide care, providing poorer quality of care to patients living with HIV compared to other patients, disclosure of HIV status without consent, physical and verbal abuse, among others [10].

In healthcare settings, individual and institutional‐level factors contribute to HIV‐related stigma and discrimination [11]. Among healthcare workers (HCWs), HIV‐related stigma is associated with a lack of knowledge about HIV transmission, concerns about occupational infection, prejudice towards KPs and certain risk behaviours, and lack of awareness about discrimination, its manifestations and consequences [11, 12]. At the institutional level, a lack of appropriate policies and protocols aimed to protect patients and HCWs and discourage discriminatory behaviours may contribute to an environment tolerant of stigma and discrimination. Interventions that focus on actionable drivers of stigma at the individual, environmental and policy levels have been shown to reduce HIV‐ and KP‐related stigma in healthcare settings [10, 12]. Such interventions have not yet been implemented widely in a low‐middle income country like Vietnam.

In Vietnam, there are an estimated 250,000 people living with HIV, a significant proportion of whom belong to KP groups, including people who inject drugs, men who have sex with men, transgender women and female sex workers [13]. In 2020, the country continued to make progress towards achieving the UNAIDS 95‐95‐95 target with 85% of people living with HIV knowing their HIV status, 78% of people who know their status on treatment and 96% of people on treatment with viral load less than 1000 copies/ml [14]. Challenges remain, however, particularly in case finding, linkage and retention, where HIV‐ and KP‐associated stigma creates barriers for patients to engage and remain in care [15].

In 2017, the Vietnam Authority of HIV/AIDS Control of the Ministry of Health launched an effort to reduce stigma and discrimination in health facilities, where people living with HIV access treatment services. A multi‐pronged facility‐level intervention was developed to identify and address actionable drivers of stigma (Table 1). We evaluated the effect of the intervention on HIV‐related stigma and discrimination among HCWs and patients receiving HIV care at healthcare facilities in three high burden provinces of Vietnam.

**Table 1:** Components of intervention to reduce stigma and discrimination in health facilities in Vietnam

Activity	Description
Introductory meeting	Review project goals and activities, gain commitment from facility leadership, ensure all stakeholders understand roles and responsibility within the project
Pre‐intervention assessment	Conduct survey on stigma and discrimination among patients and health workers from facility
Data review and activity planning workshop	Provide feedback on results of assessment to each facility, facilitate discussion of data among facility leaders, health workers, PLHIV and community leaders, perform root cause analysis and co‐design interventions to address identified actionable drivers of stigma and discrimination
Participatory training	Conduct 2‐day training of facility health workers on HIV‐ and KP‐related stigma and discrimination with 11 modules: Opening activities (expectations and objectives) Naming stigma and discrimination through pictures How stigma feels (reflection) Naming stigma and discrimination in our health facility Homework: true/false questions about key populations Testimonies by key populations The blame game Value clarification (debate) Fear‐based stigma and discrimination and universal precautions Analysing stigma and discrimination in our health facility Action planning
Recognizing champions	Host ceremony with certificates provided to key opinion leaders, both HCWs and patients, within each facility who championed stigma reduction efforts
Review and revision of facility policies	Review, revision and dissemination of facility policies discouraging discrimination and reinforcing rights of PLHIV within health facilities
Information, education and communication activities	Use regular health worker staff meetings to disseminate policy updates, provide brief education sessions on universal precautions and risk of HIV transmission, and facilitate PLHIV testimonials. Sharing of information on social media pages and posters at facilities
PLHIV and KP engagement	Involve PLHIV and KP leaders in all aspects of the project, including data collection, training, workshops, intervention design, activity planning and policy revision
Post‐intervention assessment	Conduct post‐intervention survey among patients and health workers from facility with timely feedback of results for continuous improvement efforts

Abbreviations: HCWs, healthcare workers; KP, key population, PLHIV, people living with HIV.

## 2 METHODS

We assessed HIV‐related stigma and discrimination among patients and HCWs at the targeted sites at baseline and 9 months following the start of the intervention. A repeated measures design was used to assess the change in stigma and discrimination among HCWs; the same HCWs were recruited for the baseline and the post‐assessments. A repeated cross‐sectional design was used to assess the change in stigma experienced by people living with HIV. An independent sample of people living with HIV was recruited for the baseline and post‐assessment to represent the population at each period.

### 2.1 Study site and population

The study was conducted at 10 health facilities in three provinces of Vietnam supported by the U.S. Centers for Disease Control and Prevention (CDC) under the U.S. President's Emergency Program For AIDS Relief. The provinces (Hanoi, Thai Nguyen and Binh Duong) were selected based on perceived need, commitment of provincial leaders and diversity of their settings. Facilities were selected by the following criteria: (1) providing ART services; (2) having at least 100 HIV‐positive patients enrolled in care; (3) eligible for HIV service provision under social health insurance; and (4) newly started HIV clinical services. The latter represented greater perceived need for stigma reduction. The study population included adults, aged 18 years or older, receiving HIV care in the selected facilities for at least 6 months and HCWs at the selected facilities.

### 2.2 Study subject selection

HCWs from all departments were grouped into three categories (physicians/physician assistants, nurses/midwifes and others [e.g. lab technicians, nurse assistants, receptionists, cleaners and security guards]) and were randomly selected by each facility's planning department to be invited to participate in the study. People living with HIV in both the pre‐ and post‐assessments were invited to participate by clinic staff upon presenting for care during the assessment period. Eligible patients were recruited consecutively until the required sample size was reached.

### 2.3 Sample size

The sample size was calculated to compare two related proportions for each domain measured among HCWs, and to compare proportions from two independent samples for each domain among people living with HIV. The sample size was then adjusted for clustering effect, finite population, non‐response and staff turnover. The largest sample size among domains in each study group (622 HCWs and 496 patients) was selected, which provided adequate power to detect an intervention effect on all domains.

### 2.4 Measures

We used questionnaires previously validated [16], used [17, 18, 19, 20] and revised for appropriateness in the Vietnam context [21]. The HCW tool was originally developed as a programmatic tool for measuring stigma in diverse country settings [16]. In 2014, the tool was adapted for use in Thailand [17]. Simultaneously, Thailand developed a tool for measuring healthcare stigma among persons living with HIV. In 2016, the Thai tools were adapted for a pilot stigma reduction project in Vietnam [21]. In addition to demographic data, the questionnaires included four domains for people living with HIV, including (1) experienced discrimination, (2) internalized stigma, (3) unwanted HIV disclosure and (4) discriminatory reproductive health advice. For HCWs, the tool contained six domains, including (1) fear of HIV infection, (2) unnecessary precautions, (3) observed enacted stigma, (4) negative attitudes towards people living with HIV, (5) working with colleagues living with HIV and (6) observed discrimination against KPs (Table 2 and File S1).

**Table 2:** Description of study outcomes—composite domain indices

Domain	Numerator	Denominator
Healthcare workers		
1. Fear of HIV infection (three items)	Number of respondents who answered they would be “worried” (a little worried/worried/very worried) to any of the three items	Respondents who answered the items, excluding those who answered “non‐applicable”
2. Unnecessary precautions and measures (two items)	Number of respondents who answered “YES” to any of the two items	
3. Observed enacted stigma (two items)	Number of respondents who answered “observed” (sometimes/often/most of the times) to any of the two items	All respondents
4. Expressed negative attitudes towards PLHIV (five items)	Number of respondents who answered “strongly agree or agree” with any of the statements/items 1–4, or “strongly disagree or disagree” with statement 5	
5. Uncomfortable working with PLHIV staff (one item)	Number of respondents who answered “uncomfortable” (a little uncomfortable, uncomfortable, very uncomfortable) to the item	
6. Observed discrimination against KP (one item/KP)	Number of respondents who answered “observed” (sometimes/often/most of the times) for each KP (MSM, FSW, MSW, PWID, TGW)	
People living with HIV		
1. Experienced discrimination (10 items)	Number of respondents who answered “YES” (in the past 12 months) to any of the 10 items	Respondents who answered at least one item within the domain, excluding those who answered “non‐applicable”
2. Internalized stigma (two items)	Number of respondents who answered “YES” (in the past 12 months) to any of the two items	
3. Experienced disclosure of HIV status by health staff (two items)	Number of respondents who answered “YES” to any of the two items	
4. Experienced discriminatory reproductive health advice based on HIV status (four items)	Number of respondents who answered “YES, in the past 12 months” to any of the four items	

Abbreviations: FSW, female sex workers; KP, key population; MSM, men who have sex with men; MSW, male sex workers; PLHIV, people living with HIV; PWID, people who inject drugs; TGW, transgender women.

### 2.5 Intervention

We used a multi‐pronged facility‐level intervention following key principles for stigma reduction defined by Nyblade et al., including addressing actionable drivers of stigma, creating partnerships between affected groups and opinion leaders, and putting affected groups at the centre of the response [1]. The core intervention was an HCW training, adapted for use in Vietnam, designed to address common fears and misconceptions about HIV, educate about HIV prevention in the healthcare setting and use participatory methods to create an open dialogue about HIV‐ and KP‐related stigma (Table 1 and File S2).

### 2.6 Data collection

Baseline data collection occurred between July and October 2018 and the post‐assessment occurred between May and July 2019. Data were collected and managed using REDCap electronic data capture tools hosted at Beth Israel Deaconess Medical Center [22]. Patients were interviewed in a private room of the health facility by trained peers who used smart phones to access the online semi‐structured questionnaire. HCWs gathered in a private room in groups of 5–10 with the data collector to complete the self‐administered survey. HCWs accessed the survey through a web‐link on their own devices and submitted their responses directly into REDCap.

### 2.7 Ethical considerations

The study was approved by the Institutional Review Boards of Beth Israel Deaconess Medical Center (#2010P000334) in Boston, USA and Hanoi University of Public Health (#18‐408/DD‐YTCC) in Hanoi, Vietnam. The study protocol was reviewed and approved in accordance with the U.S. CDC human research protection procedures and was determined to be research, but CDC investigators did not interact with human subjects or have access to identifiable data or specimens for research purposes (#2018‐092a). All subjects provided written informed consent prior to participation.

### 2.8 Data analysis

The main outcomes were composite domain indices (Table 2). Proportions and 95% confidence intervals (CI) were calculated for categorical variables; and means and standard deviations (SD) for continuous variables. *T*‐test was used to compare means. McNemar's test was used to compare paired proportions before and after the intervention among HCWs, and chi‐square test was used to compare proportions before and after the intervention among people living with HIV. HCWs were included in the analysis only if they completed the baseline assessment, attended the training intervention and completed the post‐assessment. Logistic mixed models were used to compare outcomes before and after intervention, taking into account subject dependence for HCWs due to the repeated measure design, and clustering effect (healthcare facilities) for both HCWs and people living with HIV. Models were adjusted for demographic factors. Due to the high correlation between years since HIV diagnosis and years on ART, only the number of years on ART was included in the models. As there were only four patients not on ART, they were excluded from the analysis. We also examined interactions between gender or occupation of HCWs and the intervention effect. Analyses were performed using Stata/SE 14.2 (Stata Corporation, College Station, TX).

## 3 RESULTS

### 3.1 People living with HIV assessment

**Figure 1:**
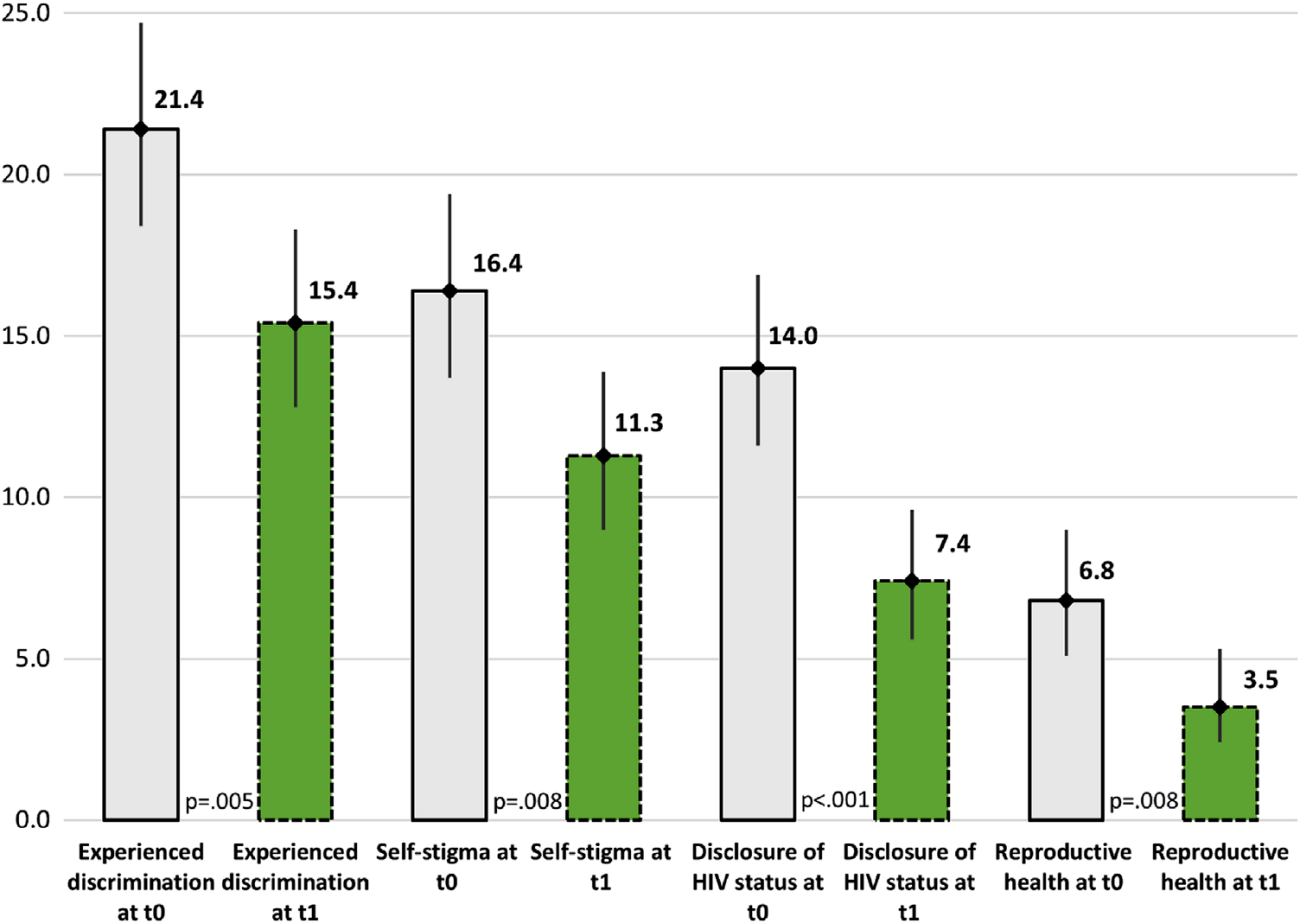
Four domains related to stigma and discrimination among people living with HIV before (t0) and after the intervention (t1), % (95% confidence interval). Note: Error bars represent 95% confidence intervals; *p*‐value determined by chi‐squared test; t0 represents the pre‐intervention assessment and t1 represents the post‐intervention assessment. This figure presents the percent of PLHIV participants who reported stigma and discrimination in each of four studied domains (experienced discrimination, internalized stigma, unwanted HIV disclosure and discriminatory reproductive health advice) at baseline and 9 months following the intervention. Abbreviation: PLHIV, people living with HIV.

Overall, 649 and 652 people living with HIV participated in the pre‐ and post‐intervention assessments, respectively. The groups were similar across age (mean age 40 years), gender (63% male), province of residence, time since HIV diagnosis, time on ART and HIV disclosure status. In the post‐intervention group, there were slightly more patients on government health insurance or reporting no insurance (Table 3). Prior to the intervention, over the previous 12 months, 21% of people living with HIV reported experiencing discrimination, 16% reported self‐stigma, 14% reported HIV disclosure without consent and 7% reported receiving discriminatory reproductive health advice. Nine months after the intervention, there was a decrease in reported stigma across all domains to 15%, 11%, 7% and 3.5%, respectively (all *p*‐values <0.05) (Figure 1). After adjusting for age, gender, time on ART, insurance status, HIV disclosure and province of residence, the odds of reporting stigma and discrimination in the post‐assessment was reduced across all domains with an adjusted OR (95% CI) of 0.64 (0.48–0.86) for experienced discrimination, 0.60 (0.43–0.84) for self‐stigma, 0.49 (0.33–0.71) for disclosure of HIV status and 0.48 (0.28–0.82) for reproductive health (Table 4).

**Table 3:** Descriptive characteristics of participating PLHIV, *n* (%) or mean ± SD

	Before *N* = 649	After *N* = 652	*p*‐value
Age (years)	39.6±8.4	39.7±8.9	0.825^a^
Gender			
Male	407 (63.1)	393 (60.3)	0.295^b^
Female	238 (36.9)	259 (39.7)	
Province			
Thai Nguyen	206 (31.7)	206 (31.6)	0.973^b^
Ha Noi	205 (31.6)	203 (31.1)	
Binh Duong	238 (36.7)	243 (37.3)	
Insurance			
Government insurance	594 (91.5)	604 (92.6)	0.001^b^
Private insurance	32 (4.9)	11 (1.7)	
No insurance	23 (3.6)	37 (5.7)	
Time from confirmed HIV (years)	8.5±4.9	7.9±5.2	0.031^a^
Disclosed HIV status			
No	100 (15.4)	122 (18.7)	0.113^b^
Yes	549 (84.6)	530 (81.3)	
Current ART			
Yes	648 (99.8)	649 (99.5)	0.624^c^
No	1 (0.2)	3 (0.5)	
Time on ART (years)	6.6±3.8	6.4±4.1	0.236^a^

Note: Four PLHIV did not report gender on the pre‐intervention assessment.

Abbreviations: ART, antiretroviral therapy; PLHIV, people living with HIV; SD, standard deviation.

^a^ *t*‐test.

^b^ chi‐squared test.

^c^ Fisher's exact test.

**Table 4:** Associations between the intervention and four domains of stigma and discrimination among people living with HIV, OR (95% CI)

	Experienced discrimination (*N* = 1284)		Self‐stigma (*N* = 1281)		Disclosure of HIV status (*N* = 1285)		Reproductive health (*N* = 1285)	
	Unadjusted	Adjusted	Unadjusted	Adjusted	Unadjusted	Adjusted	Unadjusted	Adjusted
Intervention								
	1	1	1	1	1	1	1	1
Before	0.68 (0.51–0.90)	0.64 (0.48–0.86)	0.65 (0.47–0.90)	0.60 (0.43–0.84)	0.49 (0.34–0.71)	0.49 (0.33–0.71)	0.47 (0.28–0.80)	0.48 (0.28–0.82)
After	0.98 (0.96–1.00)	0.99 (0.97–1.00)	0.99 (0.97–1.01)	1.00 (0.98–1.02)	0.98 (0.96–1.00)	0.98 (0.95–1.00)	0.98 (0.95–1.01)	0.99 (0.96–1.02)
Age Gender								
Male	1	1	1	1	1	1	1	1
Female	1.11 (0.83–1.48)	1.13 (0.84–1.53)	1.77 (1.28–2.45)	1.85 (1.33–2.58)	1.64 (1.14–2.35)	1.60 (1.10–2.31)	0.77 (0.45–1.32)	0.81 (0.47–1.41)
Disclosed HIV status								
No	1	1	1	1	1	1	1	1
Yes	0.74 (0.52–1.05)	0.75 (0.52–1.07)	0.76 (0.51–1.14)	0.81 (0.53–1.22)	0.79 (0.50–1.25)	0.76 (0.48–1.21)	0.69 (0.38–1.25)	0.72 (0.39–1.33)
Years on ART								
<5 years	1	1	1	1	1	1	1	1
5 to <10 years	0.84 (0.61–1.16)	0.87 (0.62–1.24)	0.86 (0.60–1.25)	0.77 (0.53–1.13)	1.26 (0.84–1.89)	1.12 (0.72–1.73)	0.96 (0.54–1.70)	1.14 (0.62–2.12)
10+ years	0.79 (0.54–1.16)	0.93 (0.61–1.41)	0.55 (0.33–0.91)	0.58 (0.35–0.96)	0.96 (0.58–1.60)	1.03 (0.60–1.78)	0.92 (0.44–1.93)	1.08 (0.50–2.34)
Type of insurance								
Government insurance	1	1	1	1	1	1	1	1
Private insurance	0.74 (0.31–1.78)	0.56 (0.23–1.37)	1.62 (0.74–3.51)	1.27 (0.58–2.78)	1.15 (0.44–3.02)	0.93 (0.34–2.49)	1.47 (0.53–4.04)	1.05 (0.37–2.97)
No insurance	1.11 (0.58–2.13)	1.02 (0.53–1.98)	1.91 (1.00–3.65)	1.76 (0.91–3.41)	0.61 (0.22–1.72)	0.60 (0.21–1.72)	1.59 (0.60–4.24)	1.67 (0.61–4.57)
Province								
Binh Duong	1	1	1	1	1	1	1	1
Hanoi	0.96 (0.69–1.33)	1.03 (0.72–1.48)	0.81 (0.56–1.16)	0.88 (0.60–1.31)	1.20 (0.70–2.07)	1.28 (0.75–2.17)	0.14 (0.06–0.37)	0.15 (0.06–0.39)
Thai Nguyen	0.65 (0.45–0.93)	0.71 (0.48–1.04)	0.40 (0.26–0.62)	0.44 (0.11–0.60)	1.21 (0.70–2.08)	1.34 (0.78–2.29)	0.24 (0.11–0.53)	0.25 (0.11–0.57)

Abbreviations: 95% CI, 95% confidence interval; ART, antiretroviral therapy; OR, odds ratio.

Women living with HIV were more likely to report self‐stigma (OR = 1.85, 95% CI = 1.33–2.58), and HIV disclosure by an HCW without consent (OR = 1.60, 95% CI = 1.10–2.31) than their male counterparts. There was no significant difference between genders in the other two domains. People living with HIV who were on ART for 10 years or more were less likely to report self‐stigma compared to those on ART for less than 5 years (OR = 0.58, 95% CI = 0.35–0.96). No differences based on time on ART were seen in the other three domains.

Some differences were seen across the three provinces. Compared to those in Binh Duong, patients in Thai Nguyen were less likely to report self‐stigma (OR = 0.44, 95% CI = 0.11–0.60) and those in Thai Nguyen and Hanoi were less likely to report discrimination related to reproductive health (Thai Nguyen: OR = 0.25, 95% CI = 0.11–0.57; Hanoi: OR = 0.15, 95% CI = 0.06–0.39). There were no statistically significant associations between age or HIV disclosure and any of the four domains.

### 3.2 HCW assessment

A total of 672 HCWs, who participated in the pre‐assessment, intervention and post‐assessment, were included in the analyses. Three‐quarters were females and mean age was 35 years (Table 5). More than half (57%) were nurses or midwives, 20% were physicians or physician assistants and 23% were other HCWs.

**Table 5:** Descriptive characteristics of participating health staff^a^ at baseline, *n* (%) or mean ± SD

Age (years), *n* = 672	34.5±8.2
Gender, *n* = 671	
Male	167 (24.9%)
Female	504 (75.1%)
Province, *n* = 672	
Thai Nguyen	192 (28.6%)
Ha Noi	209 (31.1%)
Binh Duong	271 (40.3%)
Occupation, *n* = 672	
Physician/physician assistant	137 (20.4%)
Nurse/midwife	382 (56.8%)
Other	153 (22.8%)
Time working at the facility (years), *n* = 659	10.6±7.8
Care for or interact with people living with HIV, *n* = 672	
No	198 (29.5%)
Yes	474 (70.5%)
Time interacting with people living with HIV (years), *n* = 454	8.8±6.7

Abbreviations: SD, standard deviation.

^a^ Baseline data, except for time working at the facility and time interacting with people living with HIV, which were not available in the baseline data.

**Figure 2:**
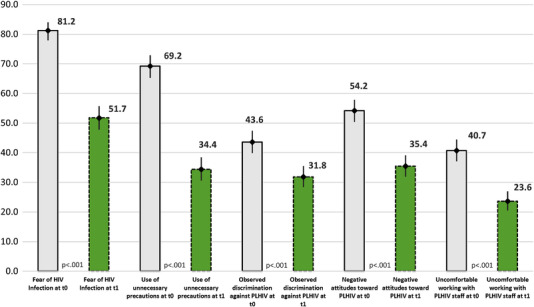
Five domains related to stigma and discrimination among healthcare workers before (t0) and after the intervention (t1), % (95% confidence interval). Note: Error bars represent 95% confidence intervals; *p*‐value determined by McNemar's test; t0 represents the pre‐intervention assessment and t1 represents the post‐intervention assessment. This figure presents the percent of healthcare worker participants who reported stigma and discrimination in each of five studied domains (fear of HIV infection, unnecessary precautions, observed enacted stigma, negative attitudes towards people living with HIV and working with colleagues living with HIV) at baseline and 9 months following the intervention. Abbreviation: PLHIV, people living with HIV.

Prior to the intervention, 81% of HCWs reported having some fear of HIV infection, 69% reported using unnecessary precautions when caring for people living with HIV, 44% reported having observed discrimination by other staff against people living with HIV, 54% reported negative attitudes towards people living with HIV and 41% reported feeling uncomfortable working with colleagues living with HIV. After the intervention, there was a significant decrease in reported stigma and discrimination across all five domains (Figure 2).

After adjusting for age, gender, occupation, years of working, contact with people living with HIV and province, the odds of reporting stigma and discrimination among HCWs in the post‐assessment reduced significantly across all domains (Table 6), including a 49% reduction in observed discrimination, 86% reduction in fear of infection and 87% reduction in the use of unnecessary precautions.

**Table 6:** Adjusted associations between the intervention and five domains of stigma and discrimination among healthcare workers, OR (95% CI)

	Fear of infection (*N* = 613)	Use of unnecessary precautions (*N* = 556)	Observed discrimination against people living with HIV (*N* = 658)	Negative attitudes towards people living with HIV (*N* = 658)	Uncomfortable working with people living with HIV staff (*N* = 656)
Intervention					
Before	1	1	1	1	1
After	0.14 (0.09–0.20)	0.13 (0.09–0.20)	0.51 (0.39–0.67)	0.38 (0.29–0.49)	0.33 (0.25–0.45)
Age	0.95 (0.90–0.99)	1.02 (0.98–1.06)	0.96 (0.93–1.00)	1.02 (0.99–1.06)	0.93 (0.90–0.97)
Gender					
Male	1	1	1	1	1
Female	0.62 (0.36–1.04)	0.86 (0.54–1.37)	0.59 (0.39–0.89)	0.78 (0.53–1.15)	0.70 (0.45–1.09)
Occupation					
Physician/physician assistant	1	1	1	1	1
Nurse/midwife	1.39 (0.77–2.50)	2.55 (1.49–4.37)	0.51 (0.32–0.82)	1.26 (0.81–1.97)	0.64 (0.39–1.06)
Other	1.12 (0.59–2.13)	0.97 (0.53–1.76)	0.21 (0.12–0.36)	0.99 (0.61–1.59)	0.37 (0.21–0.65)
Years of working					
<5 years	1	1	1	1	1
5 to <10 years	1.16 (0.64–2.09)	1.28 (0.75–2.16)	1.28 (0.80–2.04)	0.87 (0.56–1.35)	1.16 (0.70–1.90)
10 to <20	1.08 (0.54–2.17)	1.66 (0.88–3.15)	1.66 (0.94–2.91)	0.92 (0.54–1.54)	1.39 (0.76–2.55)
20+ years	2.32 (0.71–7.52)	2.38 (0.81–7.04)	2.47 (0.96–6.35)	0.80 (0.33–1.91)	2.35 (0.84–6.54)
Contact with people living with HIV					
No	1	1	1	1	1
Yes	1.60 (1.00–2.55)	1.38 (0.88–2.17)	1.62 (1.11–2.36)	1.01 (0.72–1.43)	0.99 (0.66–1.47)
Province					
Binh Duong	1	1	1	1	1
Thai Nguyen	1.40 (0.82–2.41)	1.82 (0.65–5.09)	0.80 (0.42–1.53)	1.48 (0.87–2.50)	0.92 (0.58–1.48)
Hanoi	1.48 (0.86–2.55)	1.08 (0.38–3.03)	1.39 (0.73–2.64)	1.31 (0.77–2.24)	2.39 (1.49–3.84)

Abbreviations: 95% CI, 95% confidence interval; OR, odds ratio.

**Figure 3:**
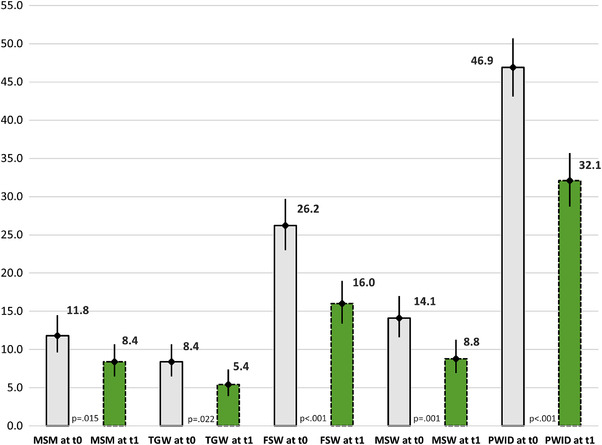
Observed discrimination against key populations among healthcare workers before (t0) and after the intervention (t1), % (95% confidence interval). Note: Error bars represent 95% confidence intervals; *p*‐value determined by McNemar's test; t0 represents the pre‐intervention assessment and t1 represents the post‐intervention assessment. This figure presents the percent of healthcare worker participants who reported having observed discrimination against key populations at baseline and 9 months following the intervention. Abbreviations: FSW, female sex workers; MSM, men who have sex with men; MSW, male sex workers; PLHIV, people living with HIV; PWID, people who injects drugs; TGW, transgender women.

Table 7 and Figure 3 present observed discrimination against KPs among HCWs. Following the intervention, reported discrimination decreased between 40% and 57% across all KP groups.

**Table 7:** Adjusted associations between the intervention and observed discrimination against key populations among healthcare workers, OR (95% CI)

	MSM (*N* = 655)	TGW (*N* = 655)	FSW (*N* = 654)	MSW (*N* = 653)	PWID (*N* = 656)
Intervention					
Before	1	1	1	1	1
After	0.60 (0.39–0.94)	0.59 (0.36–0.94)	0.45 (0.32–0.63)	0.56 (0.38–0.82)	0.43 (0.33–0.57)
Age	0.97 (0.91–1.03)	0.96 (0.91–1.02)	0.95 (0.90–0.99)	0.97 (0.92–1.02)	0.96 (0.92–1.00)
Gender					
Male	1	1	1	1	1
Female	0.55 (0.28–1.09)	0.73 (0.38–1.38)	0.93 (0.56–1.55)	1.58 (0.90–2.77)	0.70 (0.45–1.10)
Occupation					
Physician/physician assistant	1	1	1	1	1
Nurse/midwife	0.79 (0.36–1.74)	0.67 (0.32–1.40)	0.69 (0.39–1.22)	0.56 (0.31–1.03)	0.69 (0.42–1.13)
Other	0.48 (0.19–1.19)	0.55 (0.24–1.28)	0.34 (0.17–0.65)	0.35 (0.17–0.72)	0.28 (0.16–0.49)
Years of working					
<5 years	1	1	1	1	1
5 to <10 years	1.40 (0.62–3.17)	1.00 (0.47–2.16)	1.79 (1.01–3.19)	1.79 (0.97–3.29)	1.33 (0.81–2.19)
10 to <20	1.26 (0.46–3.41)	1.32 (0.53–3.32)	1.42 (0.70–2.90)	1.39 (0.65–2.99)	1.10 (0.61–2.00)
20+ years	1.41 (0.27–7.44)	1.59 (0.33–7.60)	2.42 (0.72–8.15)	1.10 (0.30–3.99)	2.21 (0.82–5.99)
Contact with people living with HIV					
No	1	1	1	1	1
Yes	1.93 (0.96–3.87)	1.44 (0.77–2.71)	2.01 (1.23–3.29)	1.58 (0.95–2.63)	1.17 (0.79–1.75)
Province					
Binh Duong	1	1	1	1	1
Thai Nguyen	0.26 (0.12–0.56)	0.42 (0.20–0.86)	0.97 (0.53–1.77)	0.34 (0.17–0.70)	1.12 (0.59–2.15)
Hanoi	0.51 (0.26–1.01)	0.92 (0.50–1.69)	2.28 (1.24–4.19)	1.72 (0.92–3.24)	2.26 (1.17–4.36)

Abbreviations: 95% CI, 95% confidence interval; FSW, female sex workers; MSM, men who have sex with men; MSW, male sex workers; OR, odds ratio; PWID, people who inject drugs; TGW, transgender women.

## 4 DISCUSSION

HIV‐ and KP‐related stigma and discrimination are well‐recognized barriers to HIV epidemic control [7, 8, 23, 24]. In Vietnam, nationwide data on stigma and discrimination in healthcare settings are generally lacking; however, available evidence suggests that stigma is pervasive across all aspects of the lives of people living with HIV. The 2014 People Living with HIV Stigma Index found that between 11% and 19% of people living with HIV avoided going to health facilities as a result of their HIV status [25]. A recent cross‐sectional study in three high‐prevalence provinces found that 86% of people living with HIV reported experiencing HIV‐related stigma, from their community (63%), family (30%) and healthcare system (8%) [26]. Our study evaluated an intervention designed to reduce HIV‐related stigma in health facilities. The results showed high rates of HIV‐ and KP‐related stigma and discrimination at baseline across all measured domains, with one out of five people living with HIV reporting having experienced discrimination in the past 12 months. Following the intervention, there were significant reductions in all measured domains.

Our intervention employed evidence‐based strategies, including engagement of facility leadership; inclusion of a broad range of health staff; use of tools and curricula adapted for local context; empowerment of key opinion leaders; and the use of participatory training methods designed to deepen HCW understanding about stigma and discrimination and its consequences, reduce fear and misconceptions about HIV transmission risk and gain commitment to act against stigma and discrimination within health facilities [11]. In addition, our approach focused on the immediate actionable drivers of stigma, and utilized quality improvement methods to empower facility leaders to use local data to tailor interventions [10, 27]. We emphasized engagement and co‐creation with the HIV‐positive and KP community, following the principle of placing communities and patients at the centre of the HIV response [28]. In addition to peer data collectors, people living with HIV and local community leaders were engaged in all aspects of the intervention, including the training, data feedback workshops and facility quality improvement teams.

It may be difficult to compare our results to other interventions previously reported as these had different approaches, timeline and measurements [29, 30]. A recent scoping review of stigma reduction interventions in healthcare settings in low‐ and middle‐income countries found that, overall, interventions to reduce HIV‐related stigma were effective in the areas they addressed and measured [31]. However, since stigma and discrimination were not defined and measured consistently, it is difficult to make meaningful comparisons across studies. Nonetheless, our study provides further evidence for the effectiveness of these strategies and highlights potential best practices to inform the design of stigma reduction programmes in other settings.

Despite finding significant reductions in all measured domains following the intervention, reported stigma and discrimination remained high in the post‐assessment. After the intervention, the proportion of HCWs reporting stigma and discrimination was above 30% in four out of five domains (Figure 2). In addition, in the post‐assessment, more than 15% of people living with HIV reported experiencing discrimination in the intervening 9‐month period. As patients often receive healthcare from more than one healthcare provider and from multiple facilities, it is possible that ongoing discrimination may have occurred at facilities that were not participating in the intervention. These findings highlight the importance of employing ongoing efforts to measure, understand and reduce HIV‐related stigma [27] and suggest that a system‐wide response may be necessary to fully tackle stigma and discrimination in the healthcare setting [32].

Our results, that 15–21% of people living with HIV experienced healthcare‐related discrimination, are comparable to those in other settings. In a pilot study of the Stigma Index 2.0, discrimination was reported by 13%, 38% and 43% of people living with HIV surveyed in Senegal, Uganda and Cameroon, respectively [33]. In contrast, in a study in South Africa and Zambia, only 7.3% reported experiencing healthcare stigma in the past year [34]. Such comparisons have limitations due to differences in measures, study population and country context, but nevertheless demonstrate the need for effective and scalable stigma reduction interventions.

Understanding HCW attitudes towards KPs and addressing intersectional stigma is crucial, particularly in a concentrated HIV epidemic as in Vietnam [4, 5, 35]. In our study, high rates of observed discrimination towards all KP groups were reported by HCWs, with particularly high rates for people who inject drugs even following the intervention. Individuals who acquired HIV through drug use suffer from intersectional stigma associated with fear of transmission as well as moral shaming of how HIV was acquired, which deters them from seeking healthcare services, disclosing their status, and contributes to unemployment, social isolation and marginalization [36]. This may be particularly true in Vietnam where HIV prevention campaigns in the early 2000s defined drug users and sex workers as “social evils” [37]. In Vietnam, stigma related to drug use has been shown to be negatively associated with access to care [38]. Likewise, men who have sex with men and transgender women in Vietnam have reported being stigmatized by the healthcare system and assert that a lack of KP‐friendly care limits their access to HIV prevention services [39, 40, 41]. Even after the intervention, we found that women living with HIV were more likely to report self‐stigma and unwanted disclosure of HIV status compared to their male counterparts after controlling for other factors. Higher rates of self‐stigma among women living with HIV have been shown in other settings, but data in Vietnam are lacking [42, 43]. Further research exploring gender issues related to stigma is needed to better inform the development of interventions focused on addressing intersectional stigma and promote KP‐friendly healthcare.

Our study may have important implications towards improving the continuum of care. Previous studies have demonstrated associations between internalized, anticipated, and experienced stigma and discrimination and outcomes along each stage of the HIV care continuum [44]. People living with HIV and members of KP groups may avoid seeking HIV testing, prevention or treatment services, or may receive inadequate quality of care if they do seek services [2]. Although we are unable to extrapolate whether the reduction in our pilot intervention translated into positive health outcomes for people living with HIV in this setting, eliminating stigma in healthcare settings is likely to improve clients’ willingness to engage in care, adherence to ART and to improve retention in care [1, 7, 8, 9, 10]. This is an important area for ongoing research.

Our study has several limitations. First, with no control group, we cannot exclude that our results are due to secular trends. Second, social desirability bias may have contributed to the improved rates of reported stigma among HCWs. It is possible that, because of the intervention, HCWs better understood about stigma and, as a result, attempted to minimize it when completing the post‐assessment. Additionally, providing the pre‐assessment results to the facilities may have created pressure to report lower stigma on the post‐assessment. However, this would not explain the concurrent decrease in stigma reported by people living with HIV in the study. Third, potential sampling bias may limit the generalizability of our results. For reasons of confidentiality and concerns about patient attrition, an independent sample of people living with HIV was enrolled for the pre‐ and post‐assessments. Moreover, patients not engaged in care or not on ART were not included in our study and may have had different experiences, which were not captured by our data. Fourth, as we did not adequately collect data on gender identity, sexual practices or injection drug use, we could not categorize individuals by KP group. As a result, KP‐related stigma was measured based on HCW observation rather than patient experience. Fifth, the post‐assessment occurred at only one time point so we cannot comment on the durability of the change. To address this and the ongoing need, we are employing routine measurement of stigma among patients and HCWs every 6–12 months. Finally, we cannot exclude potential contributions to our findings from other concurrent efforts to reduce HIV‐ or KP‐related stigma. However, given the intensity of our intervention at the participating facilities, it is unlikely that other efforts would have had a significant impact on our results.

## 5 CONCLUSIONS

Reducing HIV‐related stigma is an important part of Vietnam's effort to end the AIDS epidemic by 2030. In our study, a multi‐pronged intervention was successful at reducing HIV‐related stigma across 10 facilities in three provinces of Vietnam. Key components of the intervention included measurement of stigma and discrimination, data review and use, participatory training of HCWs, and meaningful engagement of people living with HIV and KP in the effort. Overall, our findings support the scale‐up of this intervention in Vietnam and highlight important components potentially applicable to other country programmes and settings.

## COMPETING INTERESTS

All authors declare that they have no known competing interests related to the work reported in this paper.

## AUTHORS’ CONTRIBUTIONS

All authors have read and approved the final manuscript. TMP, AN, HDC and DHT conceived the study. TMP, DTH, DTNV and DTP designed the study and wrote the study protocol. NKU, VTL, NVT, LAKA and NTN facilitated and supervised data collection. DTNV, DTP and VTTN collected the data. DTH analysed the data. TMP and DTH wrote the paper. LAC revised and HDC approved the final version of the manuscript.

## DISCLAIMER

The findings and conclusions in this report are those of the author(s) and do not necessarily represent the official position of the funding agencies.

## FUNDING

This research publication has been supported by the President's Emergency Plan for AIDS Relief (PEPFAR) through the Centers for Disease Control and Prevention (CDC) under the terms of cooperative agreement # NU2GGH002212 managed by Beth Israel Deaconess Medical Center.

## Supporting information


**File S1**: Study questionnaires.

**File S2**: Description of the intervention.

## Data Availability

The data that support the findings of this study are available from the corresponding author, TMP, upon reasonable request.
