# Supplementary material for: A pretest‐posttest design to assess the effectiveness of an intervention to reduce HIV‐related stigma and discrimination in healthcare settings in Vietnam
Source: J Int AIDS Soc. 2022 Jul 12;25(Suppl 1):e25932. doi: 10.1002/jia2.25932 (PMC9274370; doi:10.1002/jia2.25932)
Supplement: Supplementary file 1 — File S1: Study questionnaires. [file JIA2-25-e25932-s001.docx]

**Healthcare worker Questionnaire**

Participant ID (year of birth, last four digits of most commonly used mobile phone number):

[ ] [ ] [ ] [ ] [ ] [ ] [ ] [ ]

Year of Birth Last 4 digits of mobile #

Data collection date______/_____/______

**Location of Data Collection:**

**Province:**

| ⬜ 1. Thai Nguyen | ⬜ 2. Hanoi |
| --- | --- |
| ⬜ 3. Binh Duong | |

**Name of the healthcare facility:** _______________________

**Part 1: General/Personal Information**

1. How old are you currently: .......... years
2. How many years have you been working as a health care professional or hospital employee? .......... years
3. Sex  ⬜ 1. Male ⬜ 2. Female ⬜ 3. Prefer not to answer
4. What is your current position/responsibility (only choose the most important one).

| ⬜ 1. Physician | ⬜ 2. Pharmacist |
| --- | --- |
| ⬜ 3. Nurse | ⬜ 4. Medical technician |
| ⬜ 5. Nurse assistant | ⬜ 6. Front desk staff |
| ⬜ 7. Cleaning staff | ⬜ 8. Security staff |
| ⬜ 9. Other (specify…………….) |  |

1. In your current work, do you care for or interact with persons living with HIV in the hospital?

⬜ 1. Yes ⬜ 0. No (*skip to 6*)

If yes: go to Q5.1:

5.1. Have long have you cared for or interacted with persons living with HIV in the hospital? .......... years

5.2. Name of department where you have cared for or interacted with persons living with HIV in the hospital:

| ⬜ 1. HIV testing and counseling | ⬜ 2. HIV Outpatient clinic (ART) |
| --- | --- |
| ⬜ 3. OBYGN | ⬜ 4. TB clinic |
| ⬜ 5. Nurse assistant | ⬜ 6. Front desk staff |
| ⬜ 7. STIs clinic | ⬜ 8. Methadone |
| ⬜ 9. Other (specify…………….) |  |

**Part 2: Infection Control and Prevention**

1. How worried would you be about getting HIV infection if you did the followings?

| **Situation** | **Not Worried** | **A Little Worried** | **Worried** | **Very Worried** | **N/A** |
| --- | --- | --- | --- | --- | --- |
| 6.1 Touched the clothing, bedding or belongings of a patient living with HIV or AIDS patient | 0. | 1. | 2. | 3. | 99. |
| 6.2 Dressed the wounds of a patient living with HIV or AIDS patient | 0. | 1. | 2. | 3. | 99. |
| 6.3 Drew blood from a patient living with HIV or AIDS patient | 0. | 1. | 2. | 3. | 99. |

1. Do you typically do any of the following measures when providing care or services for a PLHIV or AIDS patient:

| **Situation** | **Yes** | **No** | **N/A** |
| --- | --- | --- | --- |
| 7.1 Wear double gloves | ⬜ 1. | ⬜ 0. | ⬜ 99. |
| 7.2 Use special infection control/prevention measures when taking care PLHIV or AIDS patients that you do not use with other patients | ⬜ 1. | ⬜ 0. | ⬜ 99. |

**Part 3: Health Facility Environment**

1. In the last 12 months, how often have you observed the following in your health facility?

| **Situation** | **Never** | **Sometimes** | **Often** | **Most of the Time** |
| --- | --- | --- | --- | --- |
| 8.1 Health care workers were unwilling to care for a patient living with or thought to be living with HIV. | 0. | 1. | 2. | 3. |
| 8.2 Health care workers were providing poorer quality of care to a patient living with or thought to be living with HIV than to other patients. | 0. | 1. | 2. | 3. |

1. How comfortable do the health care workers in this facility feel when having to work with co-workers or colleagues who are living with HIV?

⬜ 1. Comfortable ⬜ 2. A little uncomfortable ⬜ 3. Uncomfortable

⬜ 4. Very uncomfortable

**Part 4: Health Facility Policies**

1. In this health facility, it is not acceptable to perform the blood test for HIV without

a patient’s acknowledgement or consent.

1. Strongly agree 2. Agree 3. Disagree 4. Strongly disagree

1. In this health facility, I will get in trouble (or have negative impacts on my job) if I

discriminate against PLHIV or AIDS patients.

1. Strongly agree 2. Agree 3. Disagree 4. Strongly disagree

12. There are adequate supplies (gloves, masks, goggles, soap and water for handwashing between patients) in this health facility to help reduce my risk of becoming infected with HIV.

1. Strongly agree 2. Agree 3. Disagree 4. Strongly disagree

13. This health facility has written guidelines and/or policies to protect PLHIV or AIDS patients from discrimination.

1.Yes 0. No 99. Don’t know/not sure

**Part 5: Opinion about PLHIV**

1. What is your opinion about the following statements?

| **Statement** | **Strongly agree** | **Agree** | **Disagree** | **Strongly disagree** |
| --- | --- | --- | --- | --- |
| 14.1 Most PLHIV do not care that they could infect other people | ⬜ 1. | ⬜ 2. | ⬜ 3. | ⬜ 4. |
| 14.2 PLHIV should be ashamed about their HIV status. | ⬜ 1. | ⬜ 2. | ⬜ 3. | ⬜ 4. |
| 14.3 People get infected with HIV because they engage in irresponsible/immoral behaviors | ⬜ 1. | ⬜ 2. | ⬜ 3. | ⬜ 4. |
| 14.4 An HIV-infected woman should be sterilized  even though she doesn’t want to. | ⬜ 1. | ⬜ 2. | ⬜ 3. | ⬜ 4. |
| 14.5 Women living with HIV should be allowed to  have babies if they wish. | ⬜ 1. | ⬜ 2. | ⬜ 3. | ⬜ 4. |

**Part 6: Issues related to Key Affected Populations regardless of their HIV status**

1. In the past 12 months, how often have you observed health care workers unwilling to care for a patient who is or thought to be:

| **Situation** | **Never** | **Sometimes** | **Often** | **Most of the Time** |
| --- | --- | --- | --- | --- |
| 15.1 MSM | ⬜ 0. | ⬜ 1. | ⬜ 2. | ⬜ 3. |
| 15.2 Transgender woman | ⬜ 0. | ⬜ 1. | ⬜ 2. | ⬜ 3. |
| 15.3 Female Sex worker | ⬜ 0. | ⬜ 1. | ⬜ 2. | ⬜ 3. |
| 15.4 Male sex worker | ⬜ 0. | ⬜ 1. | ⬜ 2. | ⬜ 3. |
| 15.5 Person who injects drugs | ⬜ 0. | ⬜ 1. | ⬜ 2. | ⬜ 3. |

**PLHIV Questionnaire**

Interview date____/____/____,

Name of interviewer_________________________

**Location of Data Collection:**

**Province:**

| ⬜ 1. Thai Nguyen | ⬜ 2. Hanoi |
| --- | --- |
| **⬜** 3. Binh Duong  **Name of the healthcare facility:** | |

1. How old are you currently ………..years
2. What is your sex?

⬜ 1. Biological male ⬜ 2. Biological female ⬜ 3. Prefer not to answer

1. Currently, what type of health insurance do you hold?

| ⬜ 1. State health insurance |
| --- |
| ⬜ 2. Commercial health insurance |
| ⬜ 3. Don’t have any health insurance |

1. (The interviewer: Please choose only one option)

When did you know that you are HIV+ (through blood test result): _______/______

Month/Year

or how long have you been confirmed with HIV+: .______ years _____ months

1. Currently, are you receiving ARV drugs

⬜ 1. Yes, currently receiving

⬜ 2. Used to receive but now stopped

⬜ 3. No never received, because:

⬜ 1 Not recommended by the guideline yet (*skip to 7*)

⬜ 2 Clinically should have started, but not yet started (skip to 7)

1. *(The interviewer: Please choose only one option)*

When did you start ARV drugs: _______/_____ (Month/Year)

or how long have you received ARV drugs: ______years ____months

1. Have you disclosed your HIV positive status to the other?

| ⬜1. No, I keep it secret |
| --- |
| ⬜ 2. Yes (can answer more than one choices)  ⬜1. only to family  ⬜2. to my close friend(s) |
| ⬜3. to people in community or workplace |

**Part 2: Experience at a Health Care Facility**

1. In the last 12 months, have you avoided going to or delayed going to a health care facility near your home for HIV-specific services or general health issues/problems (not specific to HIV illness) (i.e. clinic, health care centers, primary care unit, hospitals- both public/private)?

⬜ 1. Yes, have avoided (continue to question 8.1)

⬜ 2. No, never avoid = → if female skip to question **9**

= → if male skip to question **10**

8.1 **If yes,** what was the reason to avoid going to the health care facility nearby your home (select all that applies).

| ⬜ 1. **Stigma related reasons - fear** **of disclosure of HIV status** | ⬜ 2. **Stigma related reasons – quality of** **service** | ⬜ 3. **Non-stigma related reasons** |
| --- | --- | --- |
| ⬜ 1.1 Fear of disclosure of  HIV status  ⬜ 1.2 I know someone or have family member work at the facility  ⬜ 1.3 Health facility is near my workplace, so colleagues might see me | ⬜ 2.1 Unfriendly services  ⬜ 2.1.1 Staff talk badly to me because of my HIV status  ⬜ 2.1.2 Made to wait longer than non-HIV patient/ put me at end of queue  ⬜ 2.1.3 Avoid touching me  ⬜ 2.1.4 Using double gloves  ⬜ 2.1.5 Staff stare at me or gossip about me  ⬜ 2.2 Previous negative experience  ⬜ 2.3 Afraid staff have negative attitudes toward PLHIV | ⬜ 3.1 Inconvenient, too far, no transportation  ⬜ 3.2 No money  ⬜ 3.3 Poor quality medical care/treatment, examination/diagnostic procedures, quality or selection of medications, provider knowledge, training, experience (but NOT about provider/patient interaction) Don’t trust provider’s medical knowledge  ⬜ 3.4 Wasn’t sick enough, don’t want treatment in facility, can treat myself |

1. **[ASK ONLY TO FEMALES]** Have you ever been pregnant while having HIV?

1. Yes, knew HIV positive during pregnancy

If yes, what was the outcome of the pregnancy? (Latest one if more than one)

| ⬜ 1. Abortion, at……… months of pregnancy because   \| ⬜ 1. Terminate pregnancy by own/family decision \| \| --- \| \| ⬜ 2. Terminate pregnancy by doctor advice \| \| ⬜ 3. Spontaneous abortion (naturally) \| |
| --- | --- | --- | --- |
| ⬜ 2. Went to ANC clinic and receive PMTCT (skip to 10) |
| ⬜ 3. Went to ANC clinic but not receive PMTCT (go to 10) |
| ⬜ 4. Avoid ANC (go to 9.1) |

2. Yes, but didn’t know HIV positive during pregnancy (skip to 10)

3. No (skip to 10)

- 1. **If yes,** what was the reason why you avoided going to the health care facility nearby your home (select all that applies).

| ⬜ 1. **Stigma related reasons - fear** **of disclosure of HIV status** | ⬜ 2. **Stigma related reasons - quality of service** | ⬜ 3. **Non-stigma related reasons** |
| --- | --- | --- |
| ⬜ 1.1 Fear of disclosure of HIV status  ⬜ 1.2 I know someone or have family member work at the facility  ⬜ 1.3 Health facility is near my workplace, so colleagues might see me  ⬜ 1.4 Fear that my HIV status will be disclosed if someone see that I utilize PMTCT service | ⬜ 2.1 Unfriendly services  ⬜ 2.1.1 Staff talk badly to me because of my HIV status  ⬜ 2.1.2 Made to wait Longer than non-HIV patient/ Put at end of queue  ⬜ 2.1.3 Avoid touching me  ⬜ 2.1.4 Using double gloves  ⬜ 2.1.5 Staff stare at me or gossip about me  ⬜ 2.2 Previous negative experience  ⬜ 2.3 Afraid staff have negative attitudes toward PLHIV | ⬜ 3.1 Inconvenient, too far, no Transport  ⬜ 3.2 No money  ⬜ 3.3 Poor quality medical care/treatment, examination/diagnostic procedures, quality or selection of medications, provider knowledge, training, experience (but NOT about provider/patient interaction) Don’t trust provider’s medical knowledge  ⬜ 3.4 Wasn’t sick enough, don’t want treatment in facility, can treat myself  ⬜ 3.4 There was no PMTCT service |

1. In the past 12 months, have any of the following happened to you in any health care facility because of your HIV status?

| **Situation** | **Yes** | **No** | **Don’t know/Not relevant** |
| --- | --- | --- | --- |
| 10.1 Health provider refused to attend to you or you were denied treatment | ⬜ 1. | ⬜ 0. | ⬜ 99. |
| 10.2 You were given a condition to change your behavior prior to receiving treatment (e.g. stop having sex, selling sex, using drugs, same-sex behavior or begin using contraception.) | ⬜ 1. | ⬜ 0. | ⬜ 99. |
| 10.3 Have you ever been told to come back later, put last in queue or made to wait longer than other patients? | ⬜ 1. | ⬜ 0. | ⬜ 99. |
| 10.4 Your record was marked as being HIV positive in a way that let people around you know you are living with HIV | ⬜ 1. | ⬜ 0. | ⬜ 99. |
| 10.5 Health provider talked badly, scolded or blamed you for having HIV | ⬜ 1. | ⬜ 0. | ⬜ 99. |
| 10.6 You received less care/attention than other patients | ⬜ 1. | ⬜ 0. | ⬜ 99. |
| 10.7 Health provider avoided touching your body | ⬜ 1. | ⬜ 0. | ⬜ 99. |
| 10.8 In the past 1 year, have you been admitted as an in-patient at a hospital | ⬜ 1.  To Q10.9 | ⬜ 0.  To Q12 | ⬜ 99. |
| 10.9 Your bed was marked as being HIV positive in a way that let people around know you are living with HIV. | ⬜ 1. | ⬜ 0. | ⬜ 99. |
| 10.10 You had to stay in an area designated only for HIV positive patients or people living with HIV | ⬜ 1. | ⬜ 0. | ⬜ 99. |
| 10.11 Health care provider asked you to place your hospital robe in an area/basket specifically designated for HIV positive patients due to your HIV status | ⬜ 1. | ⬜ 0. | ⬜ 99. |

1. If you answer ‘Yes’ to any of the previous situations. Where did it happen?

| ⬜ 1. At this facility |
| --- |
| ⬜ 2. At other facilities, please name: |
|  |

1. In the past 12 months, have you ever decided not to go to a health facility because of the following:

| **Situation** | **Yes** | **No** | **Don’t know** |
| --- | --- | --- | --- |
| 12.1 Feeling ashamed of your HIV status. | ⬜ 1. | ⬜ 0. | ⬜ 99. |
| 12.2 Being afraid that health facility staff will stare or gossip about you | ⬜ 1. | ⬜ 0. | ⬜ 99. |
| 12.3 Feeling guilty about your HIV status. | ⬜ 1. | ⬜ 0. | ⬜ 99. |

1. In the past 12 months, have you ever skipped or delayed taking your ARVs because of fear that other people will suspect your HIV status?

1. Yes 0. No 99. Not on ART

**Part 3: Disclosure and Confidentiality**

1. Has a health care provider ever disclosed your HIV status to other people (including husband/wife, other family members, or other people) without your consent?

⬜ 1. Yes ⬜ 0. No ⬜ 99. Not sure

1. How confidential do you think the medical records relating to your HIV status are

*(****Interviewer:*** *Read each of the choices/options below so that the respondent can select the one that applies)*

1. I am **sure** that my medical records will be kept completely confidential.

2. I am **not sure** if my medical records are confidential.

3. I feel that my medical records are **not** being kept confidential at all.

If the answer is #3, please state why you believe that your records are not being kept confidential?

**Part 4: Having Children and Reproductive Health**

Since you were diagnosed as HIV positive, have any of the incidents described below happened to you? *(****Interviewer:*** *Ask the questions below without considering the gender of the respondent-all questions can be answered by all genders).*

1. Has a healthcare provider ever advised you “not to have sex” because of your HIV status?

0. Never 1. Yes, in the past 12 months,

2 Yes, over 12 months 3. N/A

1. Has a healthcare provider ever advised you not to have children since you were diagnosed as HIV positive?

0. Never 1. Yes, in the past 12 months,

2. Yes, over 12 months 3. N/A

1. You were told that you can receive antiretroviral drugs only if you use contraception, including sterilization?

0. Never 1. Yes, in the past 12 months,

2. Yes, over 12 months 3. N/A

1. Have you / your partner (for males) ever been advised or coerced to terminate any pregnancy due to your or your partner’s (for males) HIV status?

0. Never, 1. Yes, in the past 12 months,

2. Yes, over 12 months 3. N/A

**Interviewer:** check the completeness of the questionnaire before going to the last section (Part 5 in the next page)

**Part 5: Gender and Diversity**

20. Which category do you think you belong in the following groups? (Select all that applies):

1. Transgender 4. Bisexual

2. MSM/Gay 5. Sex worker

3. Lesbian 6. Person who inject drug

7. Others (specify) …………………………………………..

*******************************
