# Supplementary material for: A pretest‐posttest design to assess the effectiveness of an intervention to reduce HIV‐related stigma and discrimination in healthcare settings in Vietnam
Source: J Int AIDS Soc. 2022 Jul 12;25(Suppl 1):e25932. doi: 10.1002/jia2.25932 (PMC9274370; doi:10.1002/jia2.25932)
Supplement: Supplementary file 2 — File S2: Description of the intervention. [file JIA2-25-e25932-s002.docx]

**Additional File 2: Intervention to Reduce Stigma and Discrimination in Healthcare Settings in Vietnam**

**Project preparation:** In December 2017, the Government of Vietnam released Directive 10 which instructed healthcare facilities to strengthen efforts to reduce HIV-related stigma and discrimination. To support healthcare facilities to implement this mandate, a project team was formed through a collaboration between the Vietnam Authority of HIV/AIDS Control (VAAC), the Partnership for Health Advancement in Vietnam (a project of the Beth Israel Deaconess Medical Center in Boston, USA), and the U.S. Centers for Disease Control and Prevention in Vietnam. The project team designed the intervention, selected the participating provinces, and reviewed, revised, and finalized the assessment tools, training materials, and other project documents.

**Introductory meeting:** An introductory meeting was conducted at each of the 3 participating provinces. The meeting included participation from the project team, the provincial centers for disease control (pCDC), and the participating health facilities. The purpose of the introductory meetings was to review the project goals and activities, gain commitment from facility leadership, and ensure all stakeholders understood the roles and responsibility within the project**.**

**Pre-intervention assessment:** The pre-intervention assessment was conducted among healthcare workers and people living with HIV receiving HIV care at the 10 participating facilities as described in the manuscript. In addition to demographic data, the assessment tools included four domains for people living with HIV (experienced discrimination, internalized stigma, unwanted HIV disclosure, and discriminatory reproductive health advice) and six domains for healthcare workers (fear of HIV infection, unnecessary precautions, observed enacted stigma, negative attitudes toward people living with HIV, discomfort working with colleagues living with HIV, and observed discrimination against key populations). The tools were adapted for the Vietnam context from tools utilized in Thailand and were piloted by the VAAC in a 2016 project in Ho Chi Minh City.

**Data review and activity planning workshop:** Facility-specific data from the pre-intervention assessment was shared with the leaders and staff of each participating facility. Workshops were organized whereby the project team facilitated a discussion of the data among facility leaders, health staff, people living with HIV, and community leaders. Workshop attendees performed a root cause analysis and co-designed activities to address the identified actionable drivers of stigma and discrimination. Examples of specific activities planned and implemented by health facilities include training of health staff in universal precautions, use of staff meetings to introduce and reinforce anti-stigma messages (such as the concept that people living with HIV who are on antiretroviral therapy with an undetectable viral load cannot transmit HIV to their sexual partners, referred to as undetectable = untransmittable or U=U), and development of codes of practice and other facility policies.

**Training of Trainers:** A 2-day training-of-trainers was organized in each of the 3 provinces for relevant staff from the pCDC and health facilities, as well as local community leaders and representatives of people living with HIV and key populations. The TOT was led by the project team and followed the training manual previously approved by the VAAC. In total, 75 people attended the three TOT courses.

**Participatory training:** Following the TOT, the main training intervention was implemented in the 10 facilities, led by the trained provincial staff with support from the project team. The 2-day training used a participatory process emphasizing discussion, group work, and other activities to encourage a process of collective learning. A mix of healthcare workers from all departments across each facility were trained in groups of 25-30 health workers per training and 2-3 trainings were organized at each facility. The 2-day training included 11 modules as listed in Table 1 of the manuscript. One module of the training included an opportunity for people living with HIV and key populations to share with health staff about their experiences and perspectives related to stigma and discrimination in the healthcare setting.

**Recognizing champions:** An awards ceremony was organized by the department of health of each province to recognize health staff and community members who made significant contributions towards stigma reduction efforts in the province. Certificates were provided to those who took specific actions following the training and according to the activities planned by each facility.

**Review and revision of facility policies:** Following the activity planning workshops and the participatory training, facility leaders were encouraged to strengthen and further disseminate policies discouraging discrimination and reinforcing rights of people living with HIV within the health facility. Each facility developed a code of practice and disseminated the policy during facility staff meetings.

**Information, education, communication activities:** Following the activity planning workshops, efforts were made to share information about the project through the facilities’ social media pages. This included general information such as the project goals and activities, reminders about universal precautions and the risk of occupational HIV transmission, as well as specific anti-stigma messaging such as U=U. U=U posters were also displayed in the HIV clinics of each facility.

**Engagement of people living with HIV and key populations**: A key principle of the intervention was to ensure meaningful involvement of community leaders in the effort. People living with HIV participated in all project activities including data collection, training, planning workshops, activity co-design and planning, and policy revision. In addition, the project encouraged collaboration between the health facilities and community groups to foster better understanding and awareness. In one participating province, a community advisory board was established as a formal collaboration mechanism. The community advisory board, consisting of key populations and individuals living with or affected by HIV in the province, interfaced with the provincial department of health and facility leaders and provided the community perspective to the design and implementation of stigma reduction activities.

**Post-intervention assessment**: Following the study design, a post-intervention assessment was conducted among patients and healthcare workers from each facility. As with the pre-intervention assessment, the project team facilitated timely feedback of the results for ongoing continuous improvement efforts.
